# A multiple classifier system identifies novel cannabinoid CB2 receptor ligands

**DOI:** 10.1186/s13321-019-0389-9

**Published:** 2019-11-07

**Authors:** David Ruano-Ordás, Lindsey Burggraaff, Rongfang Liu, Cas van der Horst, Laura H. Heitman, Michael T. M. Emmerich, Jose R. Mendez, Iryna Yevseyeva, Gerard J. P. van Westen

**Affiliations:** 1 Department of Computer Science, University of Vigo, ESEI - Escuela Superior de Ingeniería Informática, Edificio Politécnico, Campus Universitario As Lagoas s/n, 32004 Ourense, Spain; 2 CINBIO - Biomedical Research Centre, University of Vigo, Campus Universitario Lagoas-Marcosende, 36310 Vigo, Spain; 3 Multicriteria Optimization and Decision Analysis (MODA) Research Group, LIACS, Leiden University, Niels Bohrweg 1, 2333-CA Leiden, The Netherlands; 4 SING Research Group, Galicia Sur Health Research Institute (IIS Galicia Sur), SERGAS-UVIGO, Vigo, Spain; 5 School of Computer Science and Informatics, De Montfort University, The Gateway, Leicester, LE1 9BH UK; 6 Drug Discovery and Safety, LACDR, Leiden University, Einsteinweg 55, 2333 CC Leiden, The Netherlands

**Keywords:** Drug discovery, Clustering methods, Measure-guided methodology, Multiple classifier systems

## Abstract

Drugs have become an essential part of our lives due to their ability to improve people’s health and quality of life. However, for many diseases, approved drugs are not yet available or existing drugs have undesirable side effects, making the pharmaceutical industry strive to discover new drugs and active compounds. The development of drugs is an expensive process, which typically starts with the detection of candidate molecules (screening) after a protein target has been identified. To this end, the use of high-performance screening techniques has become a critical issue in order to palliate the high costs. Therefore, the popularity of computer-based screening (often called virtual screening or in silico screening) has rapidly increased during the last decade. A wide variety of Machine Learning (ML) techniques has been used in conjunction with chemical structure and physicochemical properties for screening purposes including (i) simple classifiers, (ii) ensemble methods, and more recently (iii) Multiple Classifier Systems (MCS). Here, we apply an MCS for virtual screening (D2-MCS) using circular fingerprints. We applied our technique to a dataset of cannabinoid CB2 ligands obtained from the ChEMBL database. The HTS collection of Enamine (1,834,362 compounds), was virtually screened to identify 48,232 potential active molecules using D2-MCS. Identified molecules were ranked to select 21 promising novel compounds for in vitro evaluation. Experimental validation confirmed six highly active hits (> 50% displacement at 10 µM and subsequent Ki determination) and an additional five medium active hits (> 25% displacement at 10 µM). Hence, D2-MCS provided a hit rate of 29% for highly active compounds and an overall hit rate of 52%.

## Introduction

In silico (or computational) drug discovery relies on different computer-based techniques to find a novel or improved bio-active compound, which should exhibit a strong affinity to a particular target. Although in silico screening is present in the drug development process since the beginning of 90s [1, 2], its relevance has been progressively increasing until becoming an essential part of the drug-development process. This fact was mainly motivated by (i) a significant improvement in the performance of computer systems, (ii) the introduction of novel algorithms and more expressive molecular descriptors, and (iii) the advent of large-scale public bioactivity databases [3].

Limited processing capabilities of computer systems during the 90s led to in silico screening mainly focused on (i) building simple mathematical modelling approaches (often implemented as cellular automatons) for large-scale simulations of complex systems [4], (ii) the development of large scale databases enabling researchers to easily store and access the information [2], and (iii) the design of (affinity) fingerprints as novel descriptors for similarity searches in molecular databases and QSAR analyses [5]. As computers’ performance increased, the use of simple Machine Learning (ML) classification schemes for screening purposes became popular. Concretely, the usage of support vector machines (SVM) [6, 7], Decision Trees (DT) [8], Naïve Bayes [9], K-Nearest Neighbors (KNN) [10], Artificial Neural Networks [11] and Self Organizing Maps (SOM) [12] were widely applied in the domain.

However, during the last decade the amount of public information available for screening has increased rapidly with the introduction of resources such as ChEMBL or PubChem [3, 13]. This fact had a negative impact on the performance of simple ML approaches due to their trend to build unstable classification models when handling a high volume of information. In order to improve the predictive performance, ML models were equipped with multiple layers (stacking, deep learning) or identical ML algorithms were combined (ensemble of classifiers [14]). Specifically, Lenselink et al. [15] demonstrate the suitability of using of Deep Neural Networks (DNN) [16] and Random Forests (RF) [17] methods against single ML models (such as Naïve Bayes or SVM) to predict the bioactivity of molecules. Additionally, recent work [18, 19] applied several Boosting (such as AdaBoost or MultiBoost) and Fuzzy Forest approaches to predict (i) bioactivity of molecules and (ii) toxicity of non-congeneric industrial chemicals, respectively.

The usage of above-mentioned ensembling methods contributed to significant performance improvements in the virtual screening domain. However, their introduction also brought about some important shortcomings such as: (i) the random selection of the information often used to build each inner classifier, (ii) the common usage of weak classifiers such as C4.5 or Decision Stumps to build up the classifier ensemble (although any ML classifier can be used) and, (iii) the impossibility combining different inner classifiers and configurations for them with concrete subsets of training information. These limitations are implicit to the definition of ensemble classifiers and are the key features to distinguish them against a great number of methods included in the Multiple Classifier Systems (MCS) [20] group. Wozniak et al. [20] revealed interesting features of MCS, including (i) their good performance when working in extreme situations such as scarcity of samples or information overload, (ii) their ability to outperform inner individual classifiers, (iii) the increase of the probability of finding an optimal model, and (iv) the reduction of the information (and hence the increase in the performance and speed) used to build each inner classifier. Keeping into account the above-mentioned issues we apply an MCS toolkit (called D2-MCS [21]) to increase the performance of virtual screening.

## Methods

This section evaluates the suitability of using D2-MCS and its application in drug discovery domain. It also introduces the dataset and measures used to perform the experimental protocol. Finally, the methodology performed to carry out the virtual screening process is explained in detail.

### Datasets

#### CB2 dataset

The data was gathered from ChEMBL version 22 based on UniProt accession P34972 [3]. The activity data were filtered for potential duplicates, no activity or data validity comments were allowed, and only data from binding assays with a pChEMBL value was kept. This led to 3925 compounds. Subsequently, compound fingerprints (FCFP_6) and physicochemical properties were calculated (see Additional file 1) [22]. No standardization was performed as the data was obtained from ChEMBL who include several curation steps before loading the molecules. The FCFP_6 fingerprints properties were computed using the fingerprints to properties component from Pipeline Pilot Version 2016.1.0 [23]; 2048 substructures/bits were selected based on their occurrence frequency in the data set [23]. A presence of 50% was the optimum frequency. Thereby, significant under- and over-representation were both avoided. In addition, Pipeline Pilot was also used to calculate the physicochemical properties [23]. Finally, the set was made into a binary classification set where the activity cut-off was set at a pChEMBL value > 7 for active compounds and written to a tab-delimited text file using the InChiKey as unique identifier [24]. The final set contained 1977 active compounds and 1948 inactive compounds (CB2Set, supporting information [25]). The obtained dataset includes 2133 attributes (84 physicochemical properties, 2048 chemical-structure features and the activity class) to describe 3925 compounds (instances). Table 1 shows the codification of each feature grouped by type.

**Table 1:** Feature characteristics and codification

Feature type	Feature values	No of features
Chemical substructure fingerprints	Binary	2048
Physicochemical descriptors	Discrete values	50
	Continuous values	34
Total		2132

As can be observed from Table 1 each chemical substructure is codified using a binary representation to indicate its presence (1) or absence (0) for each chemical compound. Additionally, the physicochemical descriptors consist of continuous or discrete values depending on the descriptor type and metric representation.

#### Validation dataset

The high-throughput screening (HTS) set was downloaded from the Enamine website (containing 1,834,362 compounds without class information). Molecules were standardized to make them compatible to ChEMBL data and encoded using the same feature representation as was used for the CB2 dataset (2048 chemical substructure fingerprints and 84 physicochemical descriptors). This set will be referred to as ValidationSet.

### Evaluation measures

Quite a few performance measures for assessing the accuracy and rank of different classification approaches exist in the drug discovery domain. Concretely, we select Matthews Correlation Coefficient (MCC) [26, 27] and the Positive Predictive Value (PPV) [28–30] measures due to their demonstrated ability to minimize false negatives (FN) and false positives (FP) errors respectively.

MCC is a performance measure designed for binary classifiers that can be used in the case of imbalanced datasets (the distribution of instances in the classes is uneven). MCC can be easily computed from the values of the confusion matrix results (true positives or TP, true negatives or TN, false positives or FP and false negatives or FN) by using Eq. 1.

1 $$ MCC = \frac{TP \times TN - FP \times FN}{{\sqrt {\left( {TP + FP} \right) \times \left( {FN + TN} \right) \times \left( {FP + TN} \right) \times \left( {TP + FN} \right)} }} $$

MCC is defined in the interval [− 1,1], where 1 stand for no classification errors, −1 means that all input instances were misclassified (inverse) and 0 reveals that the classification was absolutely uncorrelated with the real truth (random). As can be extrapolated from Eq. 1, achieving a balanced number of positive and negative classification hits is mandatory to obtain higher MCC values. Additionally, the inclusion of the four quantiles (TP, TN, FP and FN) in the MCC formula allows giving a better summary of the performance of classification algorithms regarding other well-known metrics (such as Accuracy [31] or F1-Score [32]). The benefits of using MCC against other well-known measures commonly used to evaluate ML approaches in the health domain has been demonstrated by Chicco [33].

From another perspective, PPV is a well-known measure in the drug discovery domain due to its ability to assess the probability of having a positive outcome given a positive classification (also called a posteriori probability). Thus, PPV is an interesting measure since testing an inactive molecule (due to an FP error) is expensive [34]. The PPV can be computed by combining values included in the confusion matrix as defined by Eq. 2.

2 $$ PPV = \frac{TP}{TP + FP} $$

As could be noted, PPV is not able to accurately handle most situations if used in isolation. In fact, a classifier could reach the maximum PPV score by identifying only one active molecule. With regard to this, over a balanced dataset where the probability of finding one active molecule is ½, a classifier could randomly select one instance to classify it as active and assign the inactive label to the remaining ones. This classifier could achieve a PPV score of one in half of the experiments (those which the instance classified as Active was really Active). Therefore, PPV needs to be accompanied by other performance indicators, such as MCC.

### Modelling

**Fig. 1:**
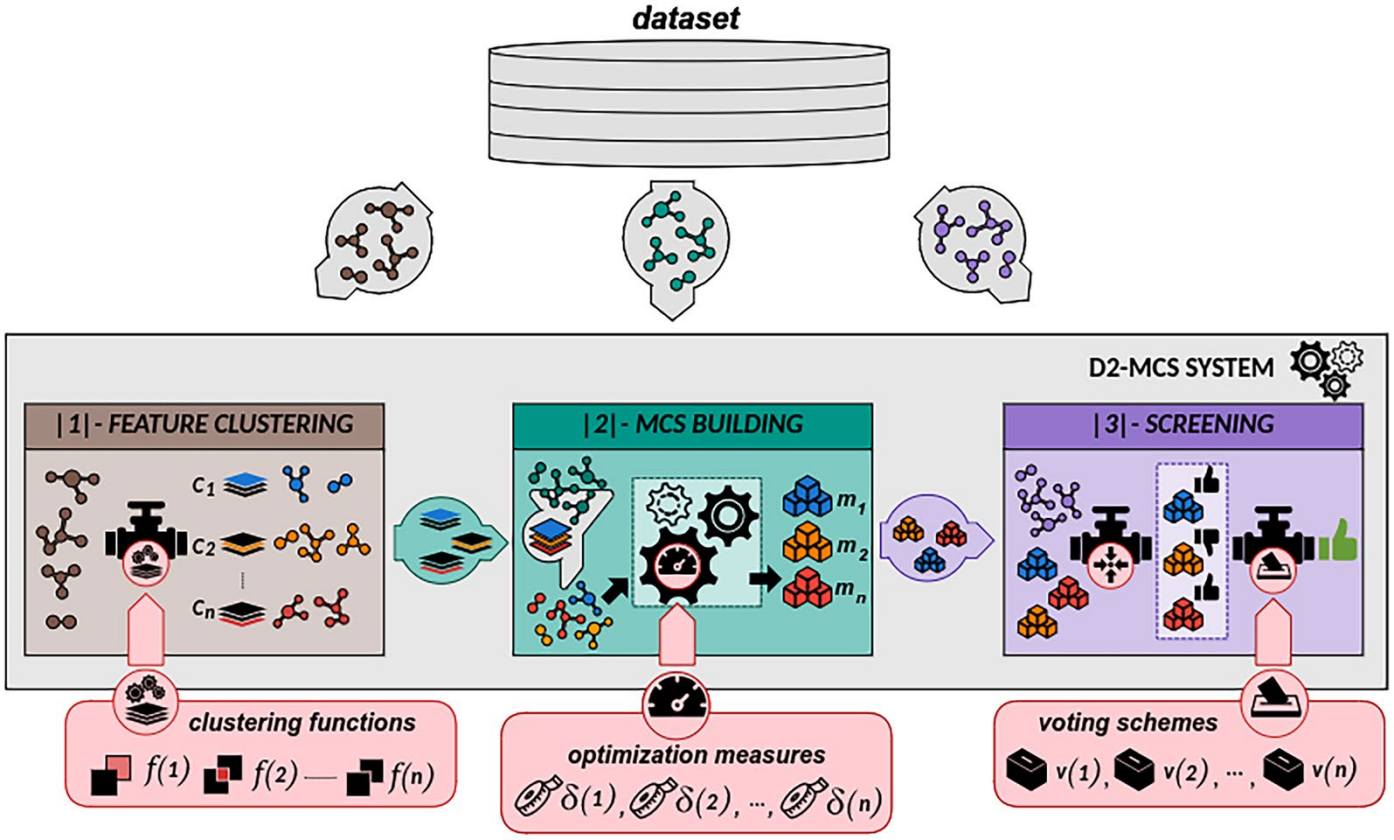
Structure and functionality of the D2-MCS toolkit. D2-MCS builds a set of classifiers (one per feature cluster) whose outputs will be combined to generate a single solution. The set of selected trained classifiers (one for each dataset part) together with a voting system comprises a whole D2-MCS instance

To build our classification software we use D2-MCS due to its ability to easily build high-performance in silico screening models [21]. D2-MCS is an R-based toolkit that provides an efficient and flexible MCS mechanism that can be highly customized to ensure an adequate adaptation to the intrinsic characteristics of the target dataset. Particularly, D2-MCS is able to handle high dimensional datasets by grouping the features of molecules (dataset columns) into several groups (called feature-clusters) according to user-defined criteria (i.e. type of chemical compounds, molecular weight, etc.). Then, for each feature-cluster, the toolkit is able to automatically determine the most suitable classifier (simple or ensemble) together with its best configuration. According to this information, D2-MCS builds a set of classifiers (one per feature cluster) whose outputs will be combined to generate a single solution. The set of selected trained classifiers (one for each dataset part) together with a voting system comprises a whole D2-MCS instance. Figure 1 shows a global overview of the D2-MCS operation.

As shown in Fig. 1, D2-MCS operation is divided into three different stages. The first stage (called FEATURE CLUSTERING in Fig. 1) comprises the partitioning of training information based on a specific feature-clustering algorithm. Although D2-MCS provides by default several clustering methods (Fisher, Information Gain, etc.), it also allows users to define customized feature clustering methods in order to increase its compatibility regardless of the way of representing or encoding the information.

During the MCS BUILDING stage, for each split of the original dataset, D2-MCS is able to detect the most effective classifier (and its best configuration) from a wide variety of ML techniques (up to 236 different classifiers from 47 families [35]). The best classifiers for each knowledge partition together with their optimal configurations are compiled together to act as a set of individual experts whose outputs should be combined to generate a final result.

Then, the third stage (see SCREENING part in Fig. 1) is the screening of molecules by combining the outputs of the classifiers selected in the previous stage. To this end, D2-MCS implements two simple methods to combine the outputs of inner classifiers and provides an API to easily define new output aggregator methods [21]. The implemented methods are: (i) a simple majority voting system where the final class is the one obtaining more than half of the votes and (ii) a weighted majority voting where the winner is the class achieving the highest overall value.

### Probabilistic-based ranking methodology

**Fig. 2:**
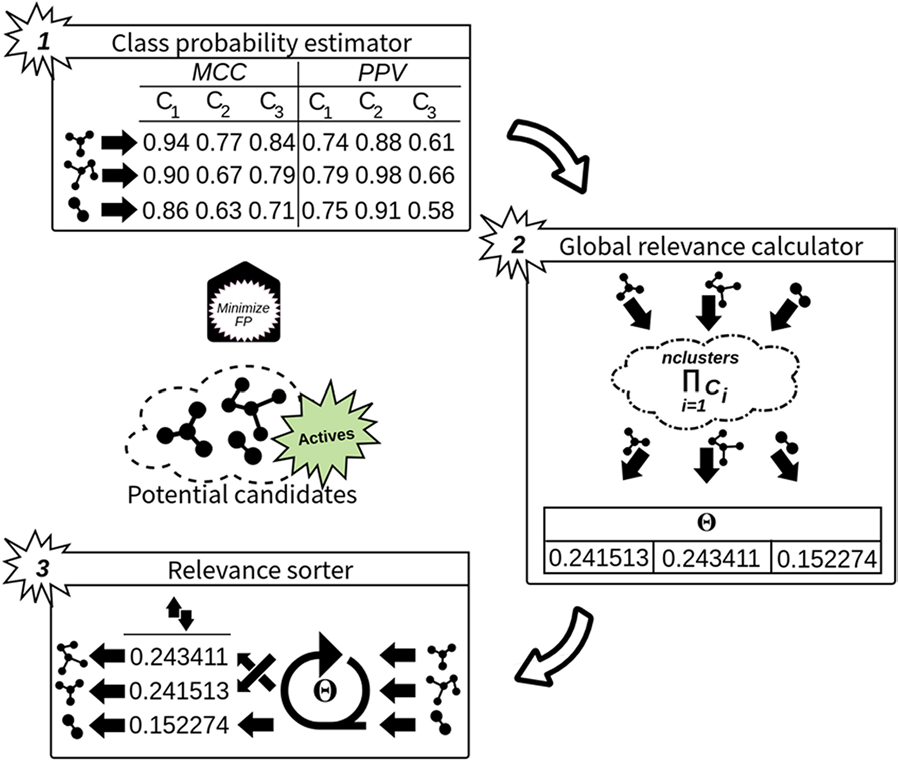
Workflow of three-stage potential candidate ranker methodology. Our ranking methodology comprised three main stages: (i) class probability estimator, (ii) global relevance calculator and (iii) relevance sorter

Due to the large number of molecules included in the validation dataset (1,834,362), the set of compounds classified as active by D2-MCS model will probably be large. A full in vitro evaluation of all these molecules is infeasible (costs, human resources, time). Hence, we designed a 3-stage probabilistic-based ranking method to select the most promising compounds from the ones receiving a positive classification. As can be seen in Fig. 2, the first stage is responsible for compiling the class probability of each compound tagged as Active (48,232) from all inner individual classifiers included in the Minimize FP model (a D2-MCS model comprising 3 classifiers for optimizing PPV and another one with 3 inner models for MCC). As can be depicted from stage 1 in Fig. 2, the achieved probabilities are always greater than 0.5 since only compounds previously labelled as Active (Active > 0.5, Inactive ≤ 0.5) were selected.

Once all probabilities are obtained, during the second stage we compute the global relevance (denoted as $\varTheta$ in Fig. 2) of each candidate as a mathematical product of all its probabilities (see Eq. 3).

3 $$ \varTheta = \prod\limits_{i = 1}^{numcluster} {C_{i} } $$

where *numcluster* stands form the number of clusters comprising the used meta-model.

Combining these probabilities using the product operator allows achieving a wide variety of output values (and thereby improves compatibility) even when individual input values are very close. As an example, given two vectors of values [0.75, 0.75, 0.6], [0.6, 0.9, 0.6], the product operator ($\varPi$) is able to achieve 0.337 and 0.324 respectively, while the summation ($\varSigma$) and the arithmetic mean ($\bar{\rm X}$) obtain the same values for both vectors (2.1 and 0.7 respectively). Finally, the third stage entails the arrangement of the chemical compounds by descendant according to its global relevance value ($\varTheta$). This ensures that the best candidates are placed in the initial positions.

### Chemical clustering

After virtual screening a further reduction of hits is required to ensure a chemically diverse set of prospective ligands for in vitro testing. Compounds identified as active by D2-MCS classifier were clustered based on the same binary features (FCFP_6) that were used for model training using the cluster molecules component in Pipeline Pilot version 2016 [23]. An average cluster population of 20 was selected and the maximum Tanimoto distance between the cluster center and members was set at 0.35 (forcing a similarity of > 0.65 within clusters). This additional requirement increases the number of clusters and thus leads to a lower number of compounds on average per cluster than the target average. However, the clusters resulting are chemically more conserved.

### In vitro experimental techniques

#### Cell culture and membrane preparation

CHOK1hCB2_bgal cells (DiscoverRx, Fremont, CA, USA) were cultured in Dulbecco’s Modified Eagle’s Medium/Nutrient Mixture F-12 Ham supplemented with 10% fetal calf serum, 1 mM glutamine, 50 µg/mL penicillin, 50 µg/mL streptomycin, 300 mg/mL hygromycin and 800 µg/mL geneticin in a humidified atmosphere at 37 °C and 5% CO_2_. Cells were subcultured twice a week at a ratio of 1:20 on 10-cm diameter plates by trypsinization. For membrane preparation, the cells were subcultured with a ratio of 1:10 and transferred to 15-cm diameter plates. The cells were collected by scraping in 5 mL phosphate-buffered saline (PBS) and centrifuged at 1000 g for 5 min. Pellets derived from 30 plates were combined and resuspended in 20 mL cold Tris–HCl, MgCl_2_ buffer (50 mM Tris–HCl (pH 7.4), 5 mM MgCl_2_). The cell suspension was homogenized using an UltraTurrax homogenizer (Heidolph Instruments Schwabach, Germany). Membranes and cytosolic fractions were separated by centrifugation in a Beckman Optima LE-80 K ultracentrifuge (Beckman Coulter Inc., Fullerton, CA, USA) at 100,000 g for 20 min at 4 °C. The supernatant was discarded. The pellet was resuspended in 10 mL cold Tris–HCl, MgCl_2_ buffer and homogenization and centrifugation steps were repeated. The membranes were resuspended in 10 mL cold Tris–HCl, MgCl_2_ buffer. Aliquots of 50 µL were stored at -80 °C until further use. The protein concentration was determined using the Pierce™ BCA Protein Assay Kit (ThermoFisher Scientific, Waltham, MA, USA).

#### [^3^H]CP55940 Displacement assay

[^3^H]CP55940 displacement assays on 96-well plates were performed in 50 mM Tris–HCl (pH 7.4), 5 mM MgCl2, and 0.1% BSA assay buffer. Membrane aliquots of CHOK1CB2_bgal containing 1.5 µg membrane protein were incubated at 25 °C for 2 h in the presence of ~ 1.5 nM [3H]CP55940 (specific activity 149 Ci/mmol; PerkinElmer, Waltham, MA). At first, all compounds were tested at a final concentration of 10 µM. When radioligand displacement was greater than 50%, full curves were recorded to determine the affinity (pKi) values of the compounds. Six different concentrations of the compounds were added by an HP D300 digital dispenser (Tecan Group Ltd, Männedorf, Switzerland). In order to determine the total binding, a control without test compound was included. Nonspecific binding was determined in the presence of 10 µM AM630. The total assay volume was 100 µL. The final concentration of DMSO was ≤ 0.25%. The incubation was terminated by rapid vacuum filtration through GF/C 96-well filter plates (PerkinElmer, Waltham, MA), to separate the bound and free radioligand, using a PerkinElmer Filtermate-harvester (PerkinElmer, Groningen, The Netherlands). Filters were subsequently washed twenty times with ice-cold assay buffer. The filter-bound radioactivity was determined by scintillation spectrometry using a Microbeta2^®^ 2450 microplate counter (PerkinElmer, Boston, MA), after addition of 25 μl MicroScint 20 (PerkinElmer, Groningen, The Netherlands) and 3 h incubation.

#### Data analysis

All experimental data were analyzed using GraphPad Prism 7 [36]. The data were normalized to percentage specific radioligand binding, where the total binding is 100% and nonspecific binding is 0%. Nonlinear regression for one-site was used to determine the IC_50_ values from the full curve [^3^H]CP55940 displacement assays. The pKi values were obtained using Eq. 4 proposed by Cheng-Prusoff [37].

4 $$ K_{i} = \frac{{IC_{50} }}{{\left( {1 + \left( {\frac{\left[ L \right]}{{K_{D} }}} \right)} \right)}} $$

where [L] is the exact concentration [^3^H]CP55940 determined per experiment and the K_D_ is the dissociation constant of [^3^H]CP55940, which is 1.24 nM as determined by Soethoudt et al. [37]. All data were obtained from three separate experiments performed in duplicate.

## Results

This section presents the performance achieved by our method. To this end, we describe the D2-MCS configuration parameters used to generate the models. Then potential screening candidates were identified by executing the previous models over the Validation set. Consecutively, the screening candidates were ranked by executing our probabilistic-based ranking methodology. Finally, in vitro analysis was performed over the selected candidates to determine their real activity.

### D2-MCS configuration

**Fig. 3:**
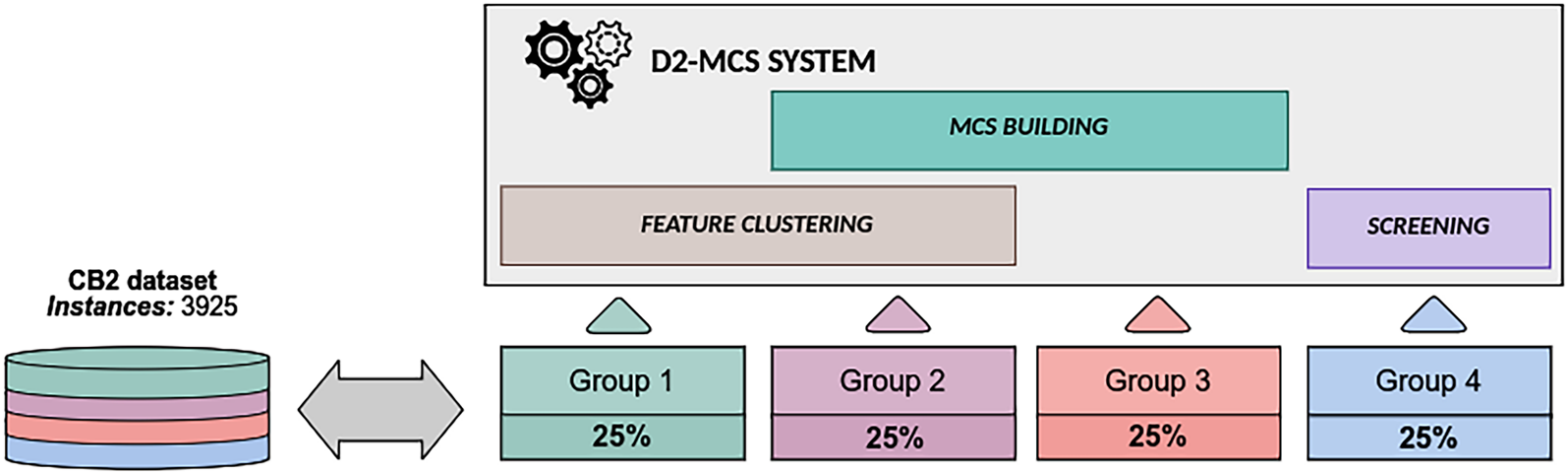
CB2 dataset partitioning. The CB2 dataset instances (rows) were randomly divided into four homogeneous and evenly sized groups

In order to execute our experimentation, the dataset instances (rows) were randomly divided into four homogeneous and evenly sized groups. Figure 3 represents the configuration of groups and their usage for: (i) executing feature clustering, (ii) building, optimizing and evaluating inner classifiers and (iii) execute a screening task for benchmark the whole D2-MCS.

As shown in Fig. 3, the first two groups were used to select an appropriate number of feature-clusters for D2-MCS. Then, the second and third groups were used to build the D2-MCS model (select the most appropriate classifier for each dataset partition, build classifiers, and optimize their configurations). Finally, the fourth group has been reserved to assess the performance of the final model.

As previously stated, during the first stage of D2-MCS process (see Fig. 1) the original dataset is divided into several groups of non-repeated features. Although the latest version D2-MCS provides several feature-clustering algorithms, we used the same clustering method as used in [21] (called *MultiTypeFisherClustering*) due to the good results achieved in this domain. Concretely, the experimentation carried out in [21] demonstrated the suitability of dividing the features into three clusters.

Once the best clustering configuration is obtained (three clusters), the D2-MCS building stage is executed. In detail, this stage is responsible for determining the best ML models (and parameter configuration) for each cluster. Additionally, D2-MCS allows defining an objective function to customize the model parameter-optimization process. To follow the same criterion as previously commented, we use both PPV and MCC measures, which entails the generation of two different D2-MCS models (PPV-based and MCC-based).

Subsequently, in order to test the final performance both obtained models (PPV-based and MCC-based) were executed over the remaining dataset (see Group 4 in Fig. 1) composed by 982 instances (504 active and 478 inactive compounds). To compute the final class of each compound, the outputs of the inner classifiers included in each D2-MCS model are combined using a voting scheme where a compound is classified as Active whenever the number of positive outputs of each inner classifier is greater or equal than the negative ones. Conversely, the compound is classified as Inactive.

**Fig. 4:**
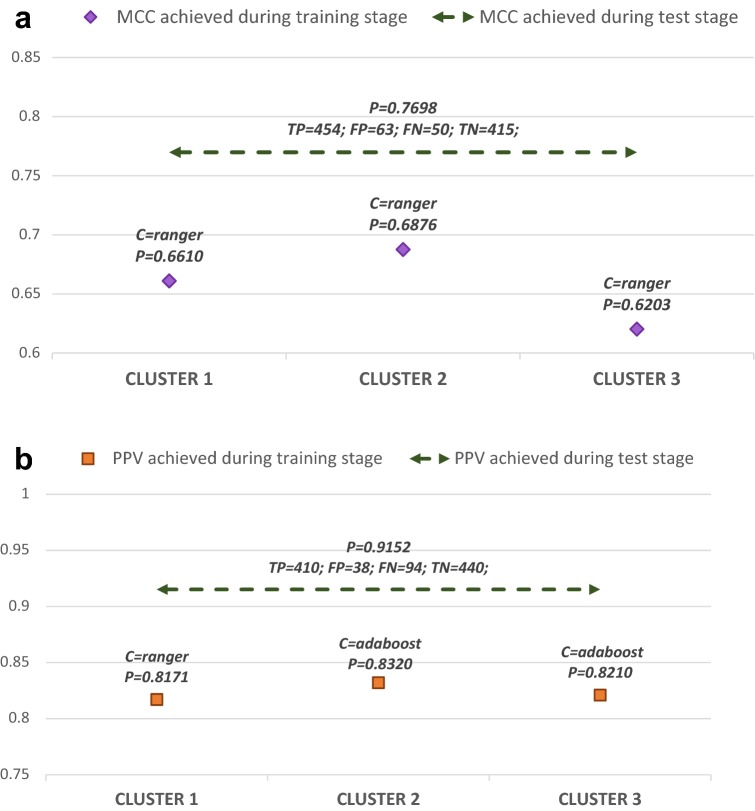
Performance comparison plot for testing stage (represented as a double arrow). **a** Indicates the performance obtained using the MCC measure and **b** shows the performance obtained using the PPV measure. Also shown (represented as dots) the name of classifier achieving the best performance for each cluster (C) together with its performance value during the optimization/training stage (P)

Following the same evaluation criteria used during the optimization stage, classification performance achieved in MCC (Fig. 4a) and PPV (Fig. 4b) scenarios were assessed using the same metric (MCC and PPV respectively). For each experimental configuration, we plotted a horizontal double arrow representing the final performance achieved during the test of the D2-MCS classifier. Additionally, for each cluster, we represent (as points) the performance achieved by the best classifier during the optimization/training stage. The graphical representation of D2-MCS performance (testing stage) also includes the numeric value represented (P) and the achieved confusion matrix (TP, TN, FP, and FN). Furthermore, the information about the best classifier for each cluster (optimization/training stage) specifies the numeric value represented (P) and the (greatest) classifier name (C).

As shown in Fig. 4a, the performance achieved during the test stage slightly outperforms the individual outcomes obtained during the optimization stage. Additionally, the use of the MCC measure allows achieving a balanced number of misclassification errors (FP ≈ FN). Furthermore, from Fig. 4b it is easy to realize that using PPV as an objective function reduces the number of FP errors at expenses of increasing FN errors. Moreover, the D2-MCS classifier achieved better performance than simple ML models (see Additional file 2).

Additionally, after performing a global overview of Fig. 4 it can be conclude that: (i) D2-MCS can be used to build suitable measure-guided knowledge-generalization models, and (ii) it is important to use an adequate domain-oriented measure in order to minimize the number of misclassification errors. In fact, as can be seen in Fig. 4, MCC based models achieve fewer error rates than the PPV measure (113 and 132 errors respectively). Despite this, the results are quite promising (the rate of correctly classified compounds is very high), although we are aware that can be increased even more by taking advantage of the intrinsic characteristics of D2-MCS.

**Fig. 5:**
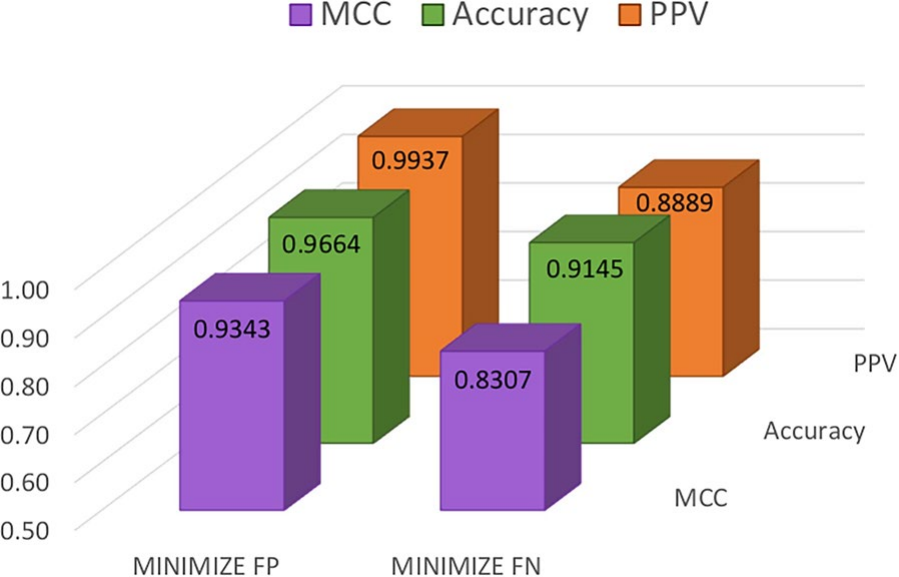
Performance comparison achieved for Minimize FP and Minimize FN meta-models

In order to demonstrate this hypothesis, we generate two meta-models by combining the predictions achieved by the D2-MCS models trained using MCC and PPV measures (see Minimize FP and Minimize FN in Fig. 5). Concretely, Minimize FP is responsible for labeling the target compound as Active whenever is predicted as ‘Active’ by both D2-MCS models (PPV and MCC) while Minimize FN identifies the target compound as Active only if one of the D2-MCS models (PPV and MCC) predicts the compound as ‘Active’. For comparison purposes, both meta-models were executed over the same testing dataset (see Group 4 in Fig. 3) as used by primitive D2-MCS models (PPV-based and MCC-based).

As can be seen in Fig. 5, both meta-models clearly improve the performance achieved by the primitive D2-MCS models. Focusing on the first approximation (Minimize FP), the performance is increased up to 21.3% (MCC) and 8.5% (PPV) regarding the original models optimized for MCC and PPV respectively. On the other hand, the second meta-model outperforms up to 7.9% (MCC) but decreases 2.9% (PPV) compared to the corresponding primitive models. The first approximation seems the most suitable alternative (best values of MCC, Accuracy, and PPV. The main reason for this circumstance can be easily explained through the confusion matrix described in Table 2.

**Table 2:** Confusion matrix achieved for both configurations

	TP	FP	TN	FN
Minimize FP	474	3	475	30
Minimize FN	480	60	418	24

As can be seen in Table 2, the number of overall errors achieved by second approximation is bigger than *Minimize FP* (84 vs 33 respectively). Considering that Accuracy computes the overall probability of performing a correct classification, it is easy to conclude that the low rate of misclassification errors motivates the good Accuracy level achieved by first approximation.

Additionally, as can be realized from Table 2, the ability to avoid discarding potential Active compounds makes Minimize FN an adequate alternative for the research domain (where discovering the whole spectrum of potential candidate drugs is more important than minimizing trial costs). Conversely, the Minimize FP approximation achieves a significant reduction of FP errors (up to 95%) when compared with Minimize FP. This fact makes Minimize FP a suitable approximation for the pharmaceutical industry where minimizing unnecessary trial tests (reduce costs) is more important than losing potential Active candidates.

### Virtual screening

We applied our D2-MCS models in virtual screening prospectively. Here, we do not know the activities of the compounds screened a priori. Virtual screening refers to the use of computational approaches to identify chemical structures that are predicted to have particular properties. To this end, we analyzed the behavior of both meta-models (*Minimize FP* and *Minimize FN*) in a realistic scenario. We classified a list of chemical compounds included in the ValidationSet in order to determine their activity. Below, Table 4 summarizes the outcomes achieved by each model grouped by activity (Active or Inactive). As can be depicted for Table 4, the number of Active compounds predicted by *Minimize FN* is higher than *Minimize FP* (representing 9.085% and 2.629% of the whole dataset), while *Minimize FP* was able to classify more compounds as Inactive.

This scenario clearly fits the behavior described in Table 3, where *Minimize FP* trends to reduce the FP rate despite sacrificing potential Active compounds while *Minimize FN* is focused on exploring all the potential candidate compounds at expenses of increasing the number of unnecessary trials (caused by FP errors).

**Table 3:** Summary of predictions group by model

Meta-models		Predictions
Minimize FP	Minimize FN	
48,232	166,664	Active
1,786,130	1,667,698	Inactive
1,834,362	1,834,362	Total

The high amount of potential Active components (48,232) makes it unfeasible (in terms of human resources and trial cost) to perform an evaluation of all the predicted actives. Therefore, we selected the most promising candidates for experimental validation from the compounds classified as Active by *Minimize FP*. We address the importance of using an adequate candidate-selection method when dealing with a reduced set of compounds (representing only 0.083% of the potential candidates) to avoid obtaining unrepresentative information. To prevent random selection of candidates, we combined a chemical clustering method with a probabilistic-based ranking methodology. The designed probabilistic-based ranking methodology was used to rank each active-predicted compound (see Additional file 3). This ranking was subsequently used to select the most suitable candidates from chemical clusters. These clusters were constituted from the list of 48,232 predicted actives. Clustering of the predicted actives resulted in 28,217 chemical clusters. From each cluster, the top scoring member (based on the ranks generated by the probabilistic-based ranking methodology) was kept while the other cluster members were discarded. Using this rank, 21 novel and diverse compounds were purchased. The average distance in the set based on Tanimoto distance was 0.81 ± 0.11, the average probability to be active was 0.77 ± 0.02, and the average distance to the training set was 0.26 ± 0.06. Hence, it can be concluded that the set selected was internally chemically diverse, highly probable to be active, and relatively close to the training set.

#### In vitro evaluation

The affinities of the 21 purchased compounds for the human CB2 receptor were determined in a radioligand displacement assay using [^3^H]CP55940 as the radiolabeled competitor (Table 4). Six compounds were able to displace more than 50% of the radioligand at 10 uM, and were thus further characterized for their affinity, where the compound with the highest affinity was ***Z336532434*** (pKi 7.67). Moreover, 5 more compounds were able to displace > 25% of the radioligand and are considered medium hits. Taken together, we were able to obtain 11 hits from the 21 novel compounds (representing a 52% hit rate). As can be seen from Table 4, four out of these 11 are in the top five based on probability. Moreover, the top 10 compounds based on probability contained 7 out of 11 actives. We conclude that our defined probability can be a good estimator of biological activity. Most notable is compound ***Z27680708***, which was measured to have a pKi of 7.46 while the Tanimoto distance to the training set was one of the largest at 0.31.

**Table 4:** Experimentally validated compounds

Data image	IDnumber/ InChiKey	Probability	Distance To closest	pKi ± SEM or % displ.
	*Z336532434* */* *MQIUMQLPFGFWME-UHFFFAOYSA-N*	*0.82*	*0.32*	*7.67 ± 0.17*
	Z28609248 / HXJYJTXXUOYRSB-UHFFFAOYSA-N	0.81	0.29	16%
	*Z26476746* */* *VYCWCTZNPBMJFW-UHFFFAOYSA-N*	*0.80*	*0.21*	*6.54 ± 0.14*
	*Z91179667* */* *XGVYRTRSINEVTE-UHFFFAOYSA-N*	*0.78*	*0.15*	*29%*
	*Z32934509* */* *OLTBRCMQFCQBIR-UHFFFAOYSA-N*	*0.78*	*0.28*	*6.47 ± 0.02*
	*Z28357657* */* *NPRYSOPFJOGFSA-UHFFFAOYSA-N*	*0.78*	*0.34*	*6.81 ± 0.29*
	Z30007452 / VBFKBSAAMKINJD-UHFFFAOYSA-N	0.77	0.24	− 2%
	*Z27687312* */* *IHBHBQAPEZJCNM-UHFFFAOYSA-N*	*0.77*	*0.23*	*7.22 ± 0.46*
	*Z46091805* */* *QKQCBVJKUBSZOR-UHFFFAOYSA-N*	*0.76*	*0.24*	*38%*
	*Z27687279* */* *WTGACPGXOMAZFA-UHFFFAOYSA-N*	*0.76*	*0.22*	*38%*
	Z44866691 / WPWBUEOMELTWOC-FCDQGJHFSA-N	0.76	0.25	− 1%
	*Z28357392* */* *VBIMVPWQQQESTK-UHFFFAOYSA-N*	*0.76*	*0.13*	*26%*
	Z1317886912 / MEXULSRPIBCDQX-UHFFFAOYSA-N	0.76	0.28	3%
	Z44867007 / PCCXRCZRXNECAZ-JLPGSUDCSA-N	0.76	0.30	0%
	Z237484560 / LIGIHTRZFDFDAN-UHFFFAOYSA-N	0.75	0.15	− 1%
	Z223843850 / CVSSLUCDGJDGHX-UHFFFAOYSA-N	0.75	0.32	− 5%
	*Z27019562* */* *WNXCAGCQOBOQMO-UHFFFAOYSA-N*	*0.75*	*0.33*	*30%*
	Z55473655 / VDTRQSFAESBVFB-UHFFFAOYSA-N	0.75	0.26	7%
	Z2094674960 / RISCNDGLDMULEE-UHFFFAOYSA-N	0.75	0.29	0%
	Z1523102560 / IXASXIGZGJSBJT-UHFFFAOYSA-N	0.75	0.30	18%
	*Z27680708* */* *HKWXDCJIBMAAFV-UHFFFAOYSA-N*	*0.74*	*0.31*	*7.46 ± 0.32*

Shown are the structure, enamine identifier (ID number), InChIKey, assigned probability, distance to the training set, and biological activity. Biological activity is shown as pKi (with a standard error of the mean) when available or  % displacement of the radioligand by 10 μM of the compound. Identified novel hits are indicated in italic

## Conclusions

This work uses Multiple Classifier Systems (MCS) in early preclinical drug discovery. Concretely, we apply D2-MCS over a training dataset to build two measure-guided D2-MCS models (PPV and MCC). Furthermore, two meta-models (*Minimize FP* and *Minimize FN*) were generated by combining the predictions achieved by the previous D2-MCS models.

Results achieved by both meta-models show the suitability of using *Minimize FP* due to its ability to avoid FP errors (only 3 from 477). To this end, we execute *Minimize FP* over a validation dataset (comprised of 1,834,62 compounds) together with our probabilistic-based ranking methodology to obtain the 21 most promising active compounds.

We have demonstrated that an appropriate combination of D2-MCS models can be successfully used for virtual screening (to predict the biological activity of chemical structures). The identified hits were chemically diverse while similar to the training set. We were successfully able to determine a probability of biological activity, which demonstrated a predictive performance for biological activity.

Despite the promising results achieved here (being a 52% hit rate), further improvements should be addressed to increase the classification performance. Therefore, future work should be focused on two main aspects (i) dataset processing and (ii) the improvement of the D2-MCS toolkit. Regarding data quality, the detection, and removal of irrelevant, noisy, or valueless features from the input dataset should be considered. Moreover, to increase the performance of D2-MCS new and efficient feature clustering methods should be implemented.

## Supplementary information

**Additional file 1.** Physicochemical descriptors comprising CB2Set.

**Additional file 2.** Performance comparison of Simple ML models and D2-MCS.

**Additional file 3.** List of potential candidates sorted by probability of being Active.

## Data Availability

The MCS framework is available on GitHub: https://github.com/drordas/D2-MCS The data used/generated in this study is available from ChEMBL and is available here: http://doi.org/10.5281/zenodo.2677650 The predicted probabilities for the virtual screening are included as Additional file.

## Abbreviations

HTS: high-throughput screening
FP: false positives
FN: false negatives
TP: true positives
TN: true negatives
MCS: multiple classifier systems
DNN: deep neural networks
SVM: support vector machines
D2-MCS: drugs discovery for multi-clustering system
PPV: positive prediction values
MCC: Matthews Correlation Coefficient
