# Supplementary material for: A multiple classifier system identifies novel cannabinoid CB2 receptor ligands
Source: J Cheminform. 2019 Nov 7;11:66. doi: 10.1186/s13321-019-0389-9 (PMC6836644; doi:10.1186/s13321-019-0389-9)
Supplement: Supplementary file 1 — Additional file 1. Physicochemical descriptors comprising CB2Set. [file 13321_2019_389_MOESM1_ESM.docx]

Additional File 1. Physicochemical descriptors comprising CB2Set

| CMP_ATOMCOUNT_C | CMP_BONDS_DOUBLEBONDS | CMP_BONDS_STEREO_FRAC |
| --- | --- | --- |
| CMP_ATOMCOUNT_C_sp3 | CMP_BONDS_TRIPLEBONDS | CMP_BONDS_RING_FRAC |
| CMP_ATOMCOUNT_C_sp2 | CMP_NUM_ALIPHATICSINGLEBONDS | CMP_BONDS_ALIPHATIC_FRAC |
| CMP_ATOMCOUNT_C_sp | CMP_NUM_ALIPHATICDOUBLEBONDS | CMP_BONDS_ROTATABLE_FRAC |
| CMP_ATOMS_C_sp3_FRAC | CMP_NUM_HYDROGENBONS | CMP_ATOMCOUNT_HALOGENS |
| CMP_ATOMS_C_sp2_FRAC | CMP_NUM_TERMINALROTOMERS | CMP_ATOMS_C_FRAC |
| CMP_ATOMS_C_sp_FRAC | CMP_ATOMCOUNT_H_ACCEPTORS | CMP_ATOMS_H_FRAC |
| CMP_FORMALCHARGE | CMP_ATOMCOUNT_H_DONORS | CMP_ATOMS_N_FRAC |
| CMP_ATOMCOUNT_HEAVY | CMP_ATOMCOUNT_H | CMP_ATOMS_O_FRAC |
| CMP_BONDS_TOTAL | CMP_ATOMCOUNT_N | CMP_ATOMS_HETEROATOM_FRAC |
| CMP_ATOMCOUNT_HYDROGENS | CMP_ATOMCOUNT_O | CMP_ATOMS_S_FRAC |
| CMP_BONDS_EXPLICIT | CMP_ATOMCOUNT_F | CMP_ATOMS_P_FRAC |
| CMP_ATOMCOUNT_POSITIVE | CMP_ATOMCOUNT_P | CMP_ATOMS_HALOGEN_FRAC |
| CMP_ATOMCOUNT_NEGATIVE | CMP_ATOMCOUNT_S | CMP_ATOMS_OTHER_FRAC |
| CMP_BONDS_RING | CMP_ATOMCOUNT_Cl | CMP_ATOMS_STEROATOM_FRAC |
| CMP_BONDS_ROTATABLE | CMP_ATOMCOUNT_Br | CMP_ATOMS_POSITIVE_FRAC |
| CMP_BONDS_AROMATIC | CMP_ATOMCOUNT_I | CMP_ATOMS_NEGATIVE_FRAC |
| CMP_BONDS_BRIDGE | CMP_MOLECULAR_SURFACEAREA | CMP_ATOMS_H_ACCEPTOR_FRAC |
| CMP_NUM_RINGS | CMP_MOLECULAR_SASA | CMP_ATOMS_H_DONOR_FRAC |
| CMP_NUM_AROMATICRINGS | CMP_MOLECULAR_POLARSASA | CMP_RIGIDITY_INDEX |
| CMP_NUM_RINGASSEMBLIES | CMP_NUM_ALIPHATICRINGS | CMP_MOLECULAR_WEIGHT |
| CMP_NUM_CHAINS | CMP_BONDS_HEAVYATOMS | CMP_CLASS_LIPINSKI_PASS |
| CMP_NUM_CHAINASSEMBLIES | CMP_BONDS_HYDROGENS | CMP_NUM_Ro5_Violations |
| CMP_ATOMCOUNT_METALATOMS | CMP_BONDS_SINGLE_FRAC | CMP_RO3_PASS |
| CMP_BONDS_PIBONDS | CMP_BONDS_DOUBLE_FRAC | CMP_MOLECULAR_POLAR_SURFACEAREA |
| CMP_BONDS_STEREOATOMS | CMP_BONDS_TRIPLE_FRAC | CMP_MOLECULAR_POLAR_SURFACEAREA_FRAC |
| CMP_BONDS_STEREOBONDS | CMP_BONDS_AROMATIC_FRAC | CMP_FULL_MWT |
| CMP_BONDS_SINGLEBONDS | CMP_BONDS_BRIDGE_FRAC | CMP_INORGANIC_FLAG |
